# Characterization and Bio-Evaluation of the Synergistic Effect of Simvastatin and Folic Acid as Wound Dressings on the Healing Process

**DOI:** 10.3390/pharmaceutics15102423

**Published:** 2023-10-04

**Authors:** Mahmoud A. Hashem, Badriyah S. Alotaibi, Mahmoud M. A. Elsayed, Manal E. Alosaimi, Amal K. Hussein, Maram H. Abduljabbar, Kyung-Tae Lee, Hamdy Abdelkader, Mohamed A. El-Mokhtar, Ahmed H.E. Hassan, Amany A. Abdel-Rheem, Amany Belal, Mohammed S. Saddik

**Affiliations:** 1 Department of Pharmaceutics and Clinical Pharmacy, Faculty of Pharmacy, Sohag University, Sohag 82524, Egypt; mahmoud.abdelaty@pharm.sohag.edu.eg (M.A.H.); amany.abdelkader@pharm.sohag.edu.eg (A.A.A.-R.); mohammed.sherif@pharm.sohag.edu.eg (M.S.S.); 2 Department of Pharmaceutical Sciences, College of Pharmacy, Princess Nourah bint Abdulrahman University, P.O. Box 84428, Riyadh 11671, Saudi Arabia; 3 Department of Basic Health Sciences, College of Medicine, Princess Nourah bint Abdulrahman University, P.O. Box 84428, Riyadh 11671, Saudi Arabia; 4 Department of Pharmaceutics, Faculty of Pharmacy, Minia University, Minia 61519, Egypt; amal_ka@yahoo.com (A.K.H.); h.abdelkader@mu.edu.eg (H.A.); 5 Department of Pharmacology and Toxicology, College of Pharmacy, Taif University, Taif 21944, Saudi Arabia; maram.a@tu.edu.sa; 6 Department of Pharmaceutical Biochemistry, College of Pharmacy, Kyung Hee University, 26 Kyungheedae-ro, Seoul 02447, Republic of Korea; 7 Department of Life and Biomedical and Pharmaceutical Sciences, College of Pharmacy, Kyung Hee University, 26 Kyungheedae-ro, Seoul 02447, Republic of Korea; 8 Department of Pharmaceutics, College of Pharmacy, King Khalid University, P.O. Box 1882, Abha 61441, Saudi Arabia; 9 Department of Medical Microbiology and Immunology, Faculty of Medicine, Assiut University, Assiut 71515, Egypt; elmokhtarma@aun.edu.eg; 10 Department of Medicinal Chemistry, Faculty of Pharmacy, Mansoura University, Mansoura 35516, Egypt; 11 Medicinal Chemistry Laboratory, College of Pharmacy, Kyung Hee University, 26 Kyungheedae-ro, Seoul 02447, Republic of Korea; 12 Medicinal Chemistry Department, Faculty of Pharmacy, Beni-Suef University, Beni-Suef 62514, Egypt; abilalmoh1@yahoo.com; 13 Department of Pharmaceutical Chemistry, College of Pharmacy, Taif University, P.O. Box 11099, Taif 21944, Saudi Arabia

**Keywords:** simvastatin, polymeric films, wound healing, gene expression, histopathological examination

## Abstract

Wound healing is a significant healthcare problem that decreases the patient’s quality of life. Hence, several agents and approaches have been widely used to help accelerate wound healing. The challenge is to search for a topical delivery system that could supply long-acting effects, accurate doses, and rapid healing activity. Topical forms of simvastatin (SMV) are beneficial in wound care. This study aimed to develop a novel topical chitosan-based platform of SMV with folic acid (FA) for wound healing. Moreover, the synergistic effect of combinations was determined in an excisional wound model in rats. The prepared SMV-FA-loaded films (SMV-FAPFs) were examined for their physicochemical characterizations and morphology. Box-Behnken Design and response surface methodology were used to evaluate the tensile strength and release characteristics of the prepared SMV-FAPFs. Additionally, Fourier transform infrared (FT-IR), differential scanning calorimetry (DSC), X-ray diffraction pattern (XRD), and animal studies were also investigated. The developed SMV-FAPFs showed a contraction of up to 80% decrease in the wound size after ten days. The results of the quantitative real-time polymerase chain reaction (RT-PCR) analysis demonstrated a significant upregulation of dermal collagen type I (CoTI) expression and downregulation of the inflammatory JAK3 expression in wounds treated with SMV-FAPFs when compared to control samples and individual drug treatments. In summary, it can be concluded that the utilization of SMV-FAPFs holds great potential for facilitating efficient and expeditious wound healing, hence presenting a feasible substitute for conventional topical administration methods.

## 1. Introduction

**Figure 1:**
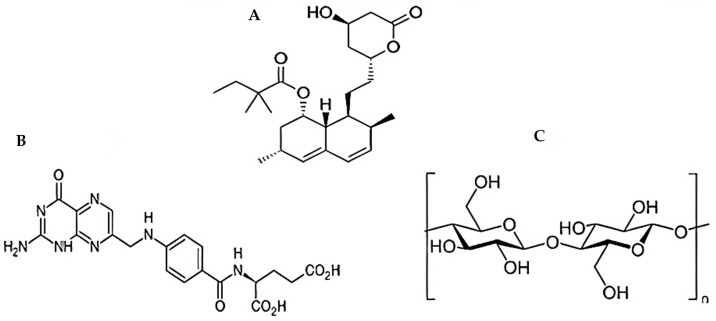
Chemical structure of (**A**) SMV, (**B**) FA, and (**C**) CHT.

Wound healing is a complex and dynamic process categorized into four distinct, sequential, and overlapping phases (hemostasis, inflammation, proliferation, and tissue remodeling) [1,2]. Vasoconstriction, thrombogenesis, angiogenesis, collagen synthesis, extracellular matrix (ECM) development, and remodeling are only a few dynamic processes during these stages [3]. Wound healing begins immediately after the injury happens and takes a variety of times to complete, depending on the severity of the injury [4]. As a result, several different medications and techniques have been widely employed to accelerate wound recovery [5]. As innovative wound-healing materials are needed, their creation becomes increasingly pressing [6]. In addition to lifestyle changes, including diet and exercise, simvastatin (SMV) is a well-known cholesterol-reducing medicine (Figure 1A) that helps lower increased lipid levels [7]. Because of this, it reduces the likelihood of having a heart attack or stroke. However, SMV has different therapeutic actions besides its cholesterol-lowering effect [8,9]. One of these is its capacity to improve mice’s impaired wound healing by increasing VEGF (Vascular Endothelial Growth Factor) synthesis and release at the wound site, a vital step in producing new blood vessels [10,11]. SMV can improve epithelialization and restore the normal skin epidermal barrier by reducing the iso-prenylation of mevalonate and farnesyl pyrophosphate (FPP downstream) targets. Decreased FPP levels can also promote keratinocyte migration in vitro, epithelialization, and wound closure in an ex vivo human culture wound healing model [12]. Wounds treated with folic acid (FA) showed markedly improved tissue regeneration (Figure 1B) due to enhanced re-epithelialization, neo-vessel formation, inflammatory cell migration, and collagen deposition [13].

The thin and flexible polymeric film, with or without a plasticizer, satisfies numerous criteria that make it an effective platform for drug release [14,15]. Polymeric films have been noticed to improve therapeutic efficacy by speeding up the initial action stage, decreasing dose frequency, and eliminating side effects [14]. Chitosan (CHT) is widely present in nature and has an antibacterial effect, heavy metal adsorption effect, antioxidation effect, and film formability [16,17,18]. They are using CHT (Figure 1C) as a polymer for manufacturing these films, which is a non-toxic, biodegradable, and biocompatible polymer [19,20]. So, CHT is considered a promising material in preparing various topical dosage forms to enhance wound healing [3,21].

An essential feature of materials used as wound dressings is their antimicrobial activity [22]. The interactions between negatively charged microbial cell membranes and positively charged CHT molecules may cause the microbial membrane to rupture, releasing proteinaceous and other intracellular constituents [23].

Considering exciting findings about the role of SMV, folic acid (FA), and CHT on wound healing, we report the novel combination of SMV and folate in CHT-based films to accelerate the healing process. Consequently, the primary goal of the current study is to formulate and characterize SMV-FAPFs for efficient wound healing. SMV-Folate loaded films were characterized for thickness, morphology, FT-IR, DSC, and XRD. An excisional wound model in rats was used to evaluate the SMV-FAPFs’ in vivo wound-healing capacity. Additionally, using the RT-PCR method, *JAK3* in skin samples and the expression of *CoTI* have been investigated.

## 2. Materials and Methods

### 2.1. Materials

Chitosan (MW∼100–300 kDa) was obtained from ACROS organics (Waltham, WA, USA). SMV was gifted by EVA pharmaceutical company and FA by El-Nile pharmaceutical company. Glycerol (≥99.5% Batch No. 360019) was procured from Lab Chem, and ethanol (CAS No. 64-17-5) was obtained from Fisher (Horsham, UK).

### 2.2. Animals and Ethical Approval

Twenty albino Rats were housed at room temperature and subjected to a light/dark cycle. Experiments were conducted per the internationally accepted Guidelines for the Care and Use of Laboratory Animals. The protocol was approved by the Faculty of Medicine Ethics Committee at Sohag University, Egypt (12/1/2022/3).

### 2.3. Methods

#### 2.3.1. Preparation of SMV-FA Polymeric Films

The solvent casting method prepared SMV-Folic acid Polymeric films (SMV-FAPFs). SMV, FA, and glycerol concentrations in all film formulations were kept constant. The solvent was prepared using a mixture of ethanol and water in different ratios (10%, 20%, and 30% *v*/*v*). SMV (100 mg) and folate (200 mg) were dissolved in 100 mL of previously prepared solutions over a stirring plate. Then, different amounts of CHT (0.5, 1, and 1.5 g) were added to the beaker with 0.1 mL of glacial acetic acid at 80 °C using a magnetic stirrer for two h, and 1 mL of glycerol was incorporated as a plasticizer. The polymer solution was sonicated for 30 min in a bath sonicator to remove bubbles in the polymeric solution, then allowed to cool to room temperature. This polymeric dispersion of the drug was poured into Teflon plates (41 cm^2^) and allowed to dry in an oven at 60 °C until a flexible film was formed. Dried films were carefully removed and checked for any imperfections or air bubbles. A funnel was placed inverted on the plate to prevent fast evaporation from the patches. Each film was cut into square batches (1 cm^2^) using a blade. The final formulations were stored in a resealable bag in a cool and dry place until use.

#### 2.3.2. Experimental Design

A full factorial (3^2^) experimental design was constructed using Stratigraphic Plus^®^ 18 software, Stat point Tech., Inc., Warrenton, VA, USA [24,25]. Three levels of each independent variable were used to optimize the prepared SMV-FAPFs [26]. The selected levels of EtOH/W ratio in total dispersion (X_1_) were 10 (−1), 20 (0), and 30% (*v*/*v*) (+1), whereas the selected values for CHT concentration in the mixture (X_2_) were 0.5 (−1), 1 (0), and 1.5 (+1) % (*w*/*v*) (Table 1). Tensile strength (TS) (Y_1_), film expansion % (Y_2_), and cumulative percent released at 3 h (Y_3_) were selected as dependent (response) parameters [27].

#### 2.3.3. Characterization of Prepared SMV-FAPFs

##### Films Expansion Profile (Expansion%)

The wound surface is simulated by a gelatin model [28], and SMV-FAPFs are placed on it. The disk-shaped films expand gradually in all directions. A clear gelatin solution was prepared by adding 4 g of gelatin powder to 100 mL of distilled water at 70 °C with constant stirring at 700 rpm. Then, 30 g of this 4% *w*/*v* gelatin solution was poured into a Petri dish and stored in the fridge overnight to form a gel. Next, each drug-loaded film was cut into a circular shape of a defined size (18 mm in diameter) and then placed on the gelatin gel (in the Petri dish). Film dressings are designed to expand after being applied to an injury and absorb wound exudate. The change in the diameter of the films was recorded after 24 h. The test was performed in duplicate for each formulation, and the mean value was used to calculate the expansion behavior using the following equation:

$$E=\frac{Dt-Do}{Do}\times 100$$

where *E* is the expansion ratio, *Dt* is the diameter of the film after expansion, and *D_o_* is the diameter of the film before expansion.

##### Tensile Strength

A universal testing device (Testometric M-500, Rochdale, Lancashire, UK) was used to measure tensile strength at the breakpoint using 3 samples cut from each film of 1.5 × 4 cm to fit into the equipment. Tensile strength was determined at 24 °C in the tension state. The instrument consisted of two tensile grips, the lower one fixed and the upper one movable. The insert formulations were positioned between two clamps. The upper arm pulled the polymeric films at a 1 mm/s rate until they broke. Tensile strength (mPa) was determined by using Exponent Lite version 6.1.4.0 (Stable Micro-System Ltd., Surrey, UK) [29].

##### In Vitro Release Study

The drug release studies from SMV-FAPFs were performed using the dissolution rate test apparatus (Hanson Research Co., Chatsworth, CA, USA). A patch of 1 cm^2^ was fixed to a circular glass slide with the help of cyanoacrylate adhesive. The pH was set to 7.4 to simulate the wound environment, and 500 mL of phosphate buffer solution and ethanol (9:1) were used as release media and deposited in a dissolving vessel. The study was conducted at 37 ± 1 °C, and paddle speed was kept at 50 rpm. Samples (5 mL) were collected for up to 6 h at suitable intervals. An aliquot of 1 mL was taken from each withdrawn sample and diluted with 1 mL of ethanol. UV-spectrophotometer measured the absorbance at λ_max_ of 238. Then, a Kinetic study was carried out to identify the release model which explains the in vitro release pattern of the drug. The release data were fitted to the Korsmeyer- Peppas equation to clarify the mechanism of SMV release from the developed film formulations [30].

$$\frac{M_{t}}{M\infty }=kt^{n}$$

where *Mt/M∞* is the fraction of drug released at time t, *k* is the kinetic constant, and *n* represents the release exponent. The value of the release exponent (*n*) determines the general release mechanism. The *n* value between 0.43 and 0.5 indicates the Fickian diffusion behavior, coupled diffusion, and polymer relaxation (non-Fickian diffusion) occurs if 0.5 < *n* < 1, erosion-mediated release (case II) takes place when *n* = 1 (zero-order kinetics, and super case II type of release, which is related to polymer relaxing and hydrogel expansion, takes place with *n* > 1 [31].

##### Thermal Analysis and Infrared Spectroscopy

Difference scanning calorimetry (DSC) tests were done on SMV, FA, CHT powder, and a physical mixture (1:1:1 *w*/*w*) on a calorimeter (DSC; PerkinElmer Thermal Analysis, Waltham, WA, USA). Three to five milligrams of a sample were sealed in a standard aluminum pan and heated over a temperature range of 25–350 °C. The thermograms were obtained at a constant heating rate of 10 °C/min in a 20 mL/min nitrogen flow rate. Fourier Transform Infrared (FTIR) analysis was applied using a spectrophotometer (Perkin Elmer Spectrum 100 FTIR Spectrometer). The FTIR spectra of the pure drug, FA, CHT, and drug-loaded films were recorded within 4000 to 550 cm^−1^.

##### X-ray Diffraction Studies

XRD has been widely used for the study of materials and thin films. XRD patterns of the pure drug, FA, polymer, physical mixture of ingredients, and SMV-loaded films are measured.

##### Microscopic Examination

Surface imaging of selected polymeric films was performed using a Scanning electron microscope. The morphology of films was determined by using a polarized microscope attached to a camera. The films were trimmed to approximately 3 cm^2^ and placed onto a glass slide covered with a slip.

##### Weight Variation

The patches were subjected to weight variation by individually weighing randomly selected patches by digital balance. Such determinations were carried out for each formulation.

##### Folding Endurance

The folding durability of polymeric films was evaluated by repeatedly folding the film strips at the same spot until the film ruptured. The polymeric films were cut into 5 cm^2^ squares. The value of folding endurance was determined by how many times the polymeric inserts could be folded at the same position without rupturing.

##### Film Thickness

The films’ thickness was measured using a digital caliper at three positions (one at the center, two near the edges). This test was performed three times for each film, and the average values were recorded.

##### Drug Content

UV-spectrophotometry was used to analyze the SMV-FAPFs for their SMV concentration. After 24 h of dissolving in 10 mL of the casting solvent, the applied film (area 1 cm^2^) was fully dissolved. After passing through a 0.45 µm filter, the solution was tested for its SMV concentration.

#### 2.3.4. Efficient Formula Selection

The most efficient SMV-FAPFs were selected based on their highest tensile strength, percentage of expansion, and cumulative percentage of release after 3 h [32]. Based on the results, F3 (EtOH/W ratio of 10% and 1.5% *w*/*v* CHT) was chosen as the best and used for further in vivo inquiry.

#### 2.3.5. In Vivo Study

##### Animal Experiments

The wound healing activities were tested in an excision wound model in rats [33,34,35,36]. Rats were housed at room temperature and subjected to a light/dark cycle. The protocols for the care and use of laboratory animals, which are universally recognized, were followed when conducting the experiments, and the protocol was approved by the Faculty of Pharmacy’s Ethics Committee (12/1/2022/3). A total of 20 albino rats were split into 4 groups of 5. The dorsal skin was shaved after intraperitoneal thiopental anesthesia, and a 1 cm^2^ circular incision was made using sterilized, sharp scissors [37,38,39]. Groups II, III, and IV received treatment twice daily with FA-loaded film, SMV-loaded film, and SMV-FAPFs 24 h after wound inoculation. Whereas the wounds of the non-treated control (group I) received blank, non-medicated films. A Canon D5200 camera (Canon, Tokyo, Japan) was used to take pictures of the wounds on days 1, 3, and 7 after the injury [6].

#### 2.3.6. Histopathological Evaluation

Skin samples were dried, embedded in paraffin, and cut into 5 μm slices after being fixed in 10% formalin on day 7 [40,41,42]. To determine the general skin structure and histological alterations in the epidermis and dermis, hematoxylin and eosin (H&E) staining was performed [43,44,45].

#### 2.3.7. CoTI and JAK3 RNA Isolation and Real-Time PCR (RT-PCR) Investigation for Skin Biopsies

RT-PCR was used to examine the expression of *CoTI* and *JAK3* in skin biopsies from treated animals to determine the wound-healing-promoting effect of SMV-FAPFs [46]. RNA was extracted from skin biopsy samples using the RNeasy Mini Kit (Qiagen, Hilden, Germany) per the manufacturer’s instructions on day seven post-treatment when a 2.5 mm needle biopsy was obtained. The high-capacity cDNA synthesis kit (Applied Biosystems, Foster City, CA, USA) was then used to create cDNA. The 7500 Fast Real-Time PCR machine (Applied Biosystems, Foster City, CA, USA) performed RT-PCR. Expression levels of *CoTI* and *JAK3* were evaluated using an evergreen based technique, and GAPDH was used as the endogenous control. Primers for the estimation of *CoTI* were: forward primer 5′-ATCAGCCCAAACCCCAAGGAGA-3′ and reverse primer 5′-CGCAGGAAGGTCAGCTGGATAG-3′, primers for the determination of JAK 3 were: *JAK3* F 5′-CCTGGAGTGGCACGAGAATC-3′, *JAK3* R 5′-TCCACAACCTCCCGCCTAT-3′ [46], while primers used for GAPDH estimation were: forward primer 5′-CCATTCTTCCACCTTTGATGCT-3′ and reverse primer 5′-TGTTGCTGTAGCCATATTCATTGT-3′ [47]. Denaturation was carried out for 10 min at 95 °C, after which a two-step cycling technique was used for 40 cycles: denaturation for 30 s at 95 °C, followed by annealing and extension for 30 s at 60 °C. RT-PCR results were analyzed using the comparative cycle threshold method (2^−ΔΔCt^ method [48]) and represented a fold-change in expression compared to the untreated control group.

## 3. Results and Discussion

### 3.1. Fabrication of SMV-FA Polymeric Film

Polymeric films were fabricated by the solvent casting method, in which the polymer is dissolved in one or more volatile solvents (organic or water) to get a homogeneous solution with a low viscosity. Nine formulations were prepared, and the composition of the investigated polymeric films is listed in Table 1. After solvent casting, the movie had smooth surfaces and was dry, thin, flexible, transparent, and free from bubbles. Tensile strength (Y_1_), film expansion % (Y_2_), and cumulative % released at 3 h (Y_3_) were selected as the response variables (Table 2).

### 3.2. DSC Studies

**Figure 2:**
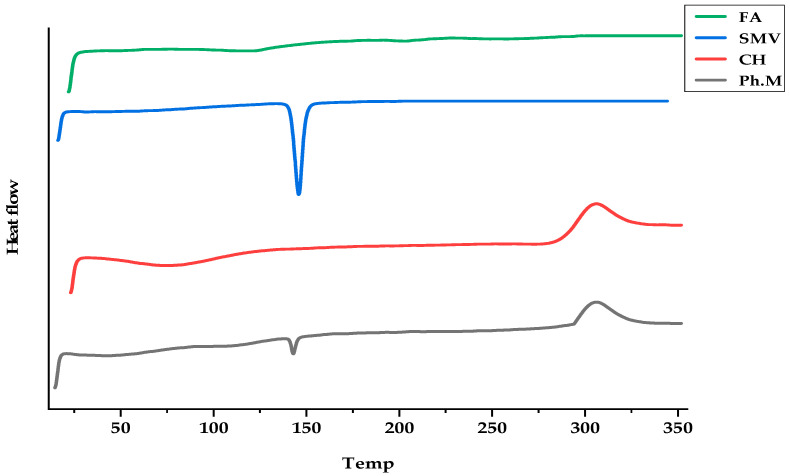
DSC thermograms of FA, SMV, CHT, and physical mixtures.

SMV, CHT, and FA interactions were studied to explain the thermal characteristics of the drug and polymer used and, hence, predict any possible physicochemical interactions that might eventually affect the drug release rate from the polymeric film formulations. The thermal curves of SMV, FA, CHT, and their physical mixture (Ph.M) are shown in Figure 2. A typical thermal behavior was recorded for SMV; a sharp endothermic peak at 145.85 °C was due to SMV melting. At the same time, FA shows two endothermic peaks at 117.6 and 203.4. The DSC thermogram of CHT showed an endothermic peak between 74.58–76.25 °C, assigned to the loss of water associated with the hydrophilic groups of CHT, and an exothermic peak at 306.22 °C, representing the degradation of acetyl and deacetylated units, and glycoside bond cleavage of CHT. The thermogram of physical mixtures of the SMV with FA and CHT showed a decrease in the intensity of the endothermic peak of SMV, indicating the phase transition of SMV into the amorphous state.

### 3.3. FTIR Investigations

**Figure 3:**
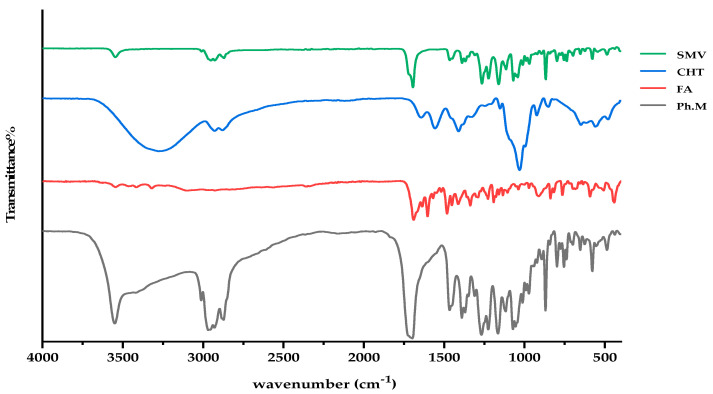
FTIR spectra of SMV, CHT, FA, and physical mixture.

Figure 3 displays the FTIR spectra of powdered mixes of SMV, FA, CHT, and their pure forms. At 3546, 2952, 2870, and 1694 cm^−1^ (stretching vibrations of O–H, C–H, C–O and C=O groups), the FTIR spectra of SMV displays characteristic peaks. Spectra of unconstrained FA show the characteristic IR absorption peaks at 1603, 1690, and 1482 cm^−1^, which are attributed to the N–H bending vibration of the O=C–NH, the C=O amide stretching of the carboxyl group, and the absorption band of the phenyl ring. In the case of CHT alone, a prominent band at 3277–3369 cm^−1^ is indicative of NH and OH stretching as well as intramolecular hydrogen bonding. No significant changes in the characteristic band positions of SMV, FA, or the spectra for CHT were found, indicating no detectable physicochemical interactions between the SMV, FA, and CHT. A possible reaction occurred between the amino group of CHT and the carboxyl group of FA, forming an amide bond. Also, the hydroxyl stretching bands got much broader in the IR of the Ph.M, indicating the formation of intermolecular hydrogen bonds between the hydroxyl groups of SMV and CHT.

### 3.4. X-ray Diffraction (XRD)

**Figure 4:**
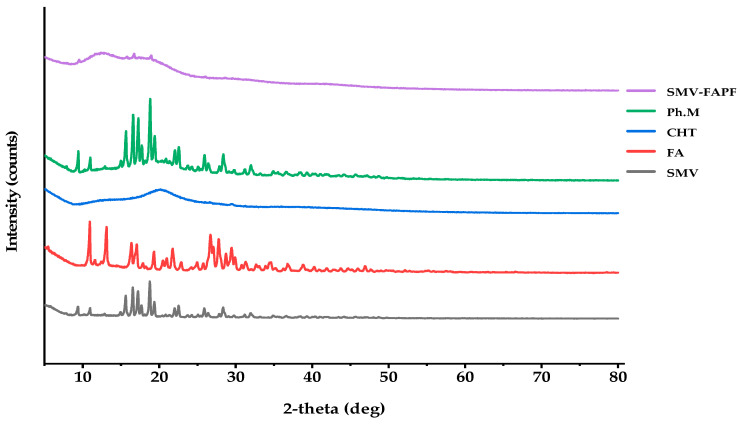
XRD for SMV-FA loaded film, physical mixture, CHT, FA, and SMV.

XRD patterns of the pure SMV, FA, CHT, SMV loaded films, and physical mixture of SMV and other excipients are presented in Figure 4. From the XRD pattern in Figure 4, pure SMV, FA, and physical mixture exhibited crystalline patterns, whereas CHT alone showed no crystalline peaks, indicating the amorphous nature of this polymer. SMV exhibited several unique sharp peaks at 9.2°, 10.9°, 15.61°, 16.52°, 17.3°, 18.84°, 19.5°, and 22.6°, which resulted from its regular crystallization. Moreover, CHT Powder peaked at 13.1°, a typical broad peak at 20.5°, whereas the diffractogram of the polymeric film showed a broad peak at 12.4°.

### 3.5. Scanning Electron Microscope

**Figure 5:**
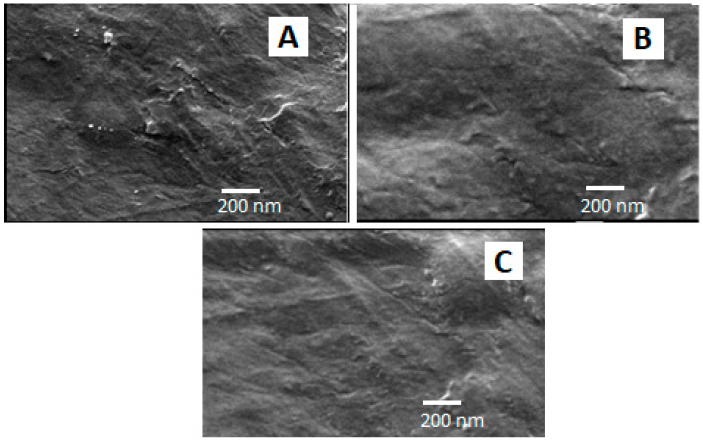
SEM micrograph of three optimized SMV-FAPFs: F1 (**A**), F2 (**B**), and F3 (**C**).

Figure 5A–C show the microscopic surface morphology of three optimized film formulations (F1, F2, and F3) in a bright field. These films also demonstrated good compatibility within the film matrix, as observed by their uniform appearance, without any phase separation or noticeable cracks.

### 3.6. Response Surface Methodology and Optimization of Formulation Factors

#### 3.6.1. Tensile Strength

**Figure 6:**
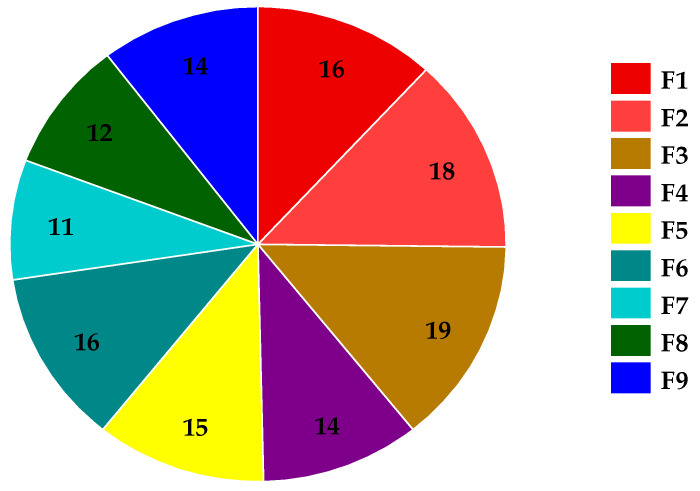
Tensile strength of SMV-FAPFs (F1–F9).

**Figure 7:**
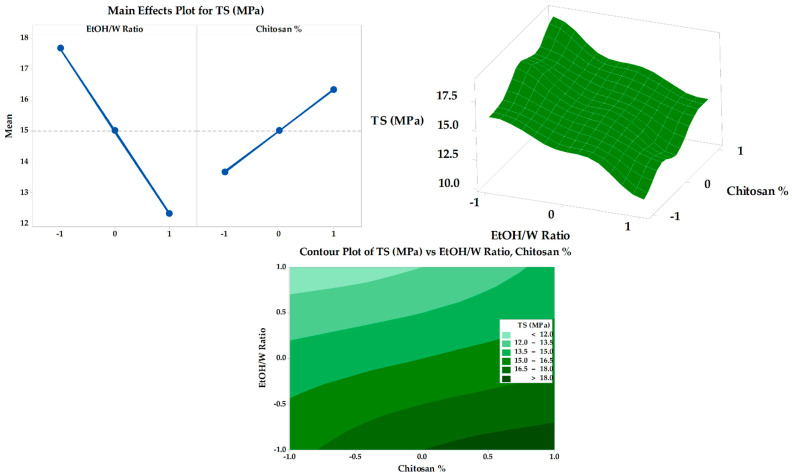
Main effects, response surface plot, and contour plot of EtOH/Ratio and CHT% on TS.

The tensile strengths of all SMV-FAPFs are represented in Figure 6. Tensile strength (TS) measures breakability parameters that determine the resistance to damage that can occur during production, handling by the patient, or placement by the clinician [6]. The TS measured in the range of 11–19 MPa. The results reported corresponded with Table 3’s folding endurance findings. Folding endurance is proportional to tensile strength. The main effects plot and response surface figure (Figure 7) showed that the SMV-FAPFs containing a lower ratio of EtOH/W have higher tensile strength than those containing higher ratios. The TS was found to be significantly affected by the ratio of EtOH/W in addition to the chitosan concentration. A high EtOH/W ratio in the polymer matrix may also produce regions of discontinuity, lowering the resistance of the matrix to fracture and leading to lower tensile strength and elastic modulus of the films. As the ratio of EtOH/W decreased, the ability of the formulated films to withstand stress increased. Also, increasing CHT concentration slightly enhanced the film’s resistance to breakage because the formation of macro-void structures became smaller and lesser, improving the film’s structure. The obtained results agreed with many studies showing that ethanol promotes the dehydration of polymer chains, increasing their intermolecular bonds and producing a stiffer material [49].

#### 3.6.2. Expansion Percentage

**Figure 8:**
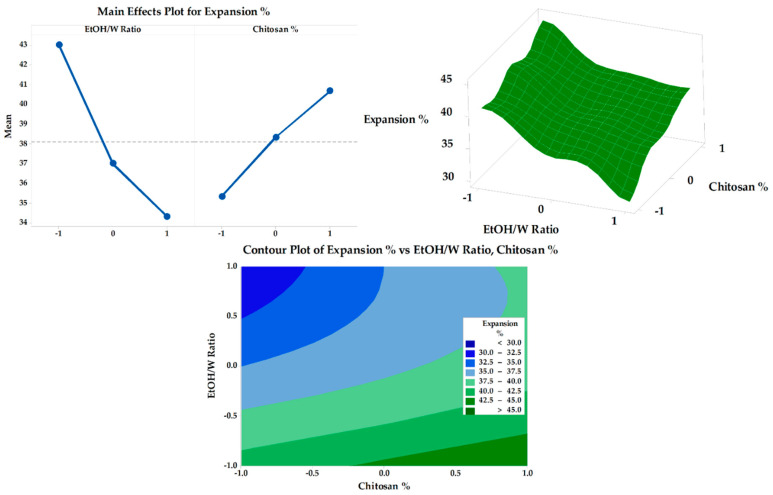
Main effects, response surface plot, and contour plot of EtOH/W ratio and CHT% on expansion%.

The obtained results of the fabricated SMV-FAPFs expansion percentage are presented in Table 2. The expansion percentage was between 30.88 ± 1.24 (F7) and 45.54 ± 1.67 (F3). The obtained result revealed a strong relationship between the EtOH/W ratio and concentration of CHT and the expansion % of the investigated SMV-FAPFs (Figure 8). A large percentage of expansion was obtained at a low EtOH/W ratio (10%). As the EtOH/W ratio increased (20% in F4, 5, and 6), the expansion percentage decreased and further decreased as the EtOH/W ratio increased to 30%. The results agree with previous studies, which explained that more hydrated films quickly absorbed more wound exudate and showed more expansion. Moreover, the concentration of CHT affected the expansion percentage. The results showed that the expansion percentage slightly increased as the CHT concentration increased. The most significant expansion percentage was recorded for F3, which has the lowest ratio of EtOH/W (10%) and the highest CHT concentration (1.5%). The expansion process included water uptake followed by the process of swelling of the polymer matrix. So, increasing CHT concentration resulted in a more significant expansion percentage, confirmed by experimental outcomes. The results showed that the expansion percentage slightly increased as the chitosan concentration increased.

#### 3.6.3. In Vitro SMV-FAPFs Release

**Figure 9:**
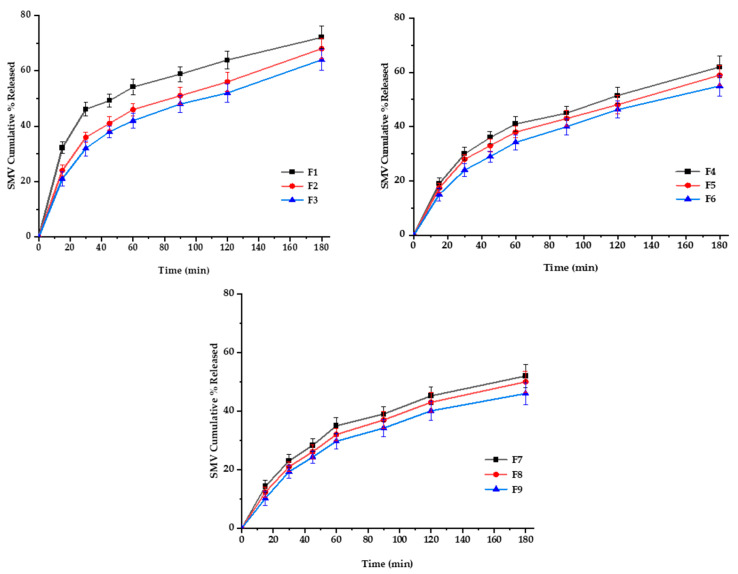
In vitro release profiles of SMV-FAPFs (F1-F9) in phosphate buffer-ethanol solution (9:1), PH 7.4, at 37 ± 0.5 °C.

**Figure 10:**
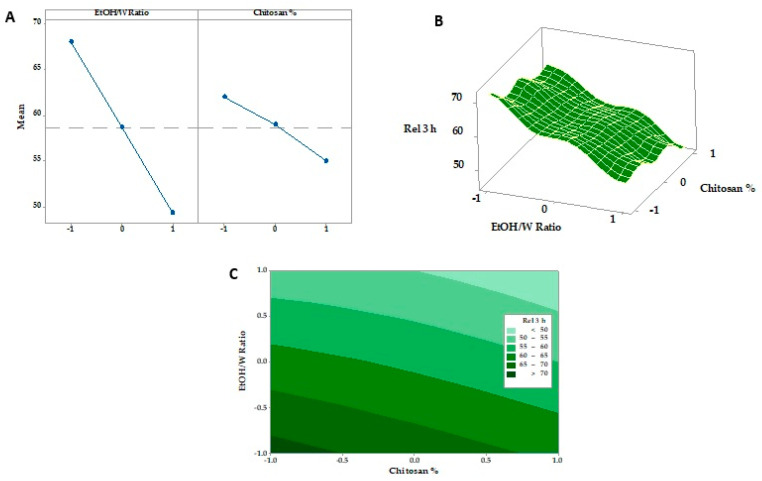
Main effects (**A**), response surface plots (**B**), and contour plot (**C**) of EtOH/Ratio and CHT % on Rel 3 h.

The diffusion rate often controls drug release from polymeric films through intact polymeric matrix or water-filled channels. Controlled drug release is due to the restricted mobility of tiny drug molecules distributed or dissolved in the dense macromolecular matrix films [50]. For SMV-FAPFs, the cumulative percentage of SMV released from the investigated formulations ranged from 46.44 ± 2.89 (F9) to 72.87 ± 2.32% (F1) after 3 h. Over 40% of SMV was released after 1 h (h) in F1, F2 and F3. In contrast, SMV cumulative % release after one h did not exceed 40% for other formulations. Other formulation did not exceed. The obtained results showed that the higher the EtOH/W ratio, the more complex the film was formed, and more time would be needed to get the film wet, resulting in a delayed SMV release. F1, prepared with a lower EtOH/W ratio, showed prompt drug release and a significantly higher value of cumulative percent release after 3 h as compared to other formulations. On the other hand, the concentration of CHT was inversely proportional to the cumulative percentage of SMV released. They were increasing the engagement of CHT results in delayed drug release due to the increment in crosslinking density [51]. Results showed that the longer the diffusion path and the more viscous the diffusion microenvironment, the slower the consequent release rate. So, when the EtOH/W ratio was constant, the formulations with low CHT concentration (F1, F4, and F7) showed enhanced SMV cumulative % released more than other formulations, as shown in Figure 9 and Figure 10. Using Korsmeyer-peppas equation, the calculated diffusional exponents, *n*, from 3 formulations with different CHT concentration (F1, F2, and F3) were 0.53, 0.56 and 0.58, respectively. All values are between 0.5 and 1, indicating that the release mechanism for the tested topical film formulations was Coupled diffusion and polymer relaxation.

### 3.7. Physical and Mechanical Characterization of the Prepared Films

The SMV-FAPFs were prepared by a simple and scalable method utilizing a mixture of water and ethanol as a solvent. The method of preparation, the amount of gel poured into the dish, and the flatness of the surface during drying all influence the thickness of the film. In this study, 20 g of gel was ideal for casting the film dressing in a plastic die with desirable properties. Films prepared from less than 20 g were so thin that they were difficult to remove from the plastic die since they were easily shredded. In contrast, thick films were unsuitable, needing more transparency and flexibility. Physical and mechanical characteristics of the optimum prepared formulae (F1, F2, and F3) according to Box-Behnken Design are shown in Table 3. Using plasticizers such as glycerin (GLY) was essential to stimulate better mechanical properties. GLY is a water-miscible liquid with a low molecular weight and a high propensity to diffuse and connect with polymer chains. The films become more malleable due to reduced stiffness and a spike in interchain distances [52]. As a result, a flexible, non-brittle, and conformable dressing for use on various wound sites has been developed. Simvastatin is a hydrophobic drug, so adding ethanol as a co-solvent enhances the solubility of the drug and gives a slight transparency to the films. 

### 3.8. In Vivo Study

**Figure 11:**
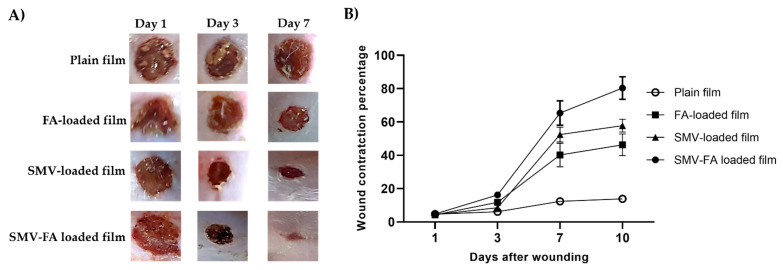
The healing progression of wounds is treated with tested preparations. (**A**) Excision wounds of rats were treated with plain film, FA-loaded film, SMV-loaded film, and SMV-FA films and photographed at 1-, 3- and 7-days post-infection to follow up the healing process. (**B**) The effect of different formulations on the percentage of wound contraction was calculated at 1, 3,7, and 10 after wounding. The results are expressed as the means ± SD.

Rats treated with SMV-FA-loaded films exhibited improved skin appearance, accelerated wound closure, and regular hair growth compared to other groups that received the other treatments at 3-and 7-days post-wounding (Figure 11A). Additionally, this group exhibited the highest wound contraction percentage (Figure 11B), pointing to a marked reduction of the wound size with enhanced healing.

### 3.9. RNA Isolation and Real-Time PCR (RT-PCR) Analysis of CoTI and JAK3 in Skin Biopsies

**Figure 12:**
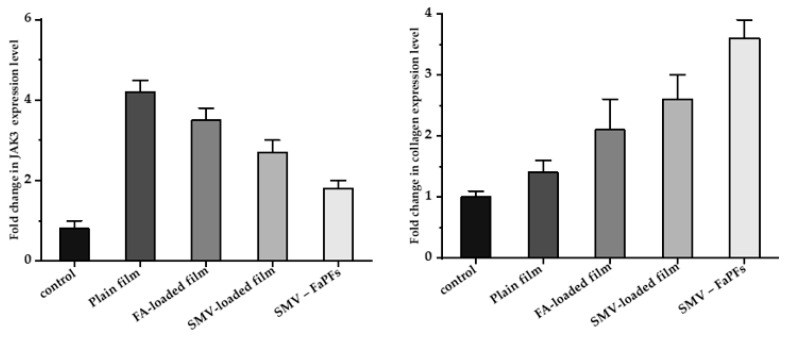
Fold expression levels of *JAK3* and *CoTI* in skin biopsies of treated rats.

*CoTI* and *JAk3* expression in the treated skin samples was assessed by RT-PCR analysis [6,53] to examine further the molecular pathways connected to SMV-FAPFs treatment, as shown in Figure 12. Generally, treated samples showed higher levels of *CoTI* and lowered *JAK3* levels. It should be noted that skin samples treated with either SMV-film or FA-film alone had considerably lower *CoTI* expression than those treated with SMV-FAPFs. Samples treated with SMV-FAPFs had a mean fold change in collagen expression of 3.6 ± 0.3, while samples treated with SMV and FAPFs had fold changes of 2.6 ± 0.4 and 2.1 ± 0.5.

Moreover, *JAK3* expression after treatment with SMV-FAPFs was downregulated compared to samples treated with SMV or FA films alone. The average fold change for *JAK3* expression was 1.8 ± 0.2 compared to 2.7 ± 0.3 and 3.5 ± 0.3 in SMV and FA-treated samples. Collagen synthesis is principally responsible for wound healing. The proliferative stage of the healing cycle is characterized by the growth of fibroblasts and the production of new collagen. The granulation layer contains collagen types I and III, with *CoTI* being the more prevalent protein there [54]. As a result, the assay of *CoTI* appears to be a valuable indicator of the wound-healing process [55]. Treatment with SMV-FAPFs was associated with a fourfold increase in *CoTI* expression, showing the preparation’s promising capacity for wound healing. Skin inflammation, linked to tissue damage, is primarily caused by the production of several immune modulators known as cytokines. Many substances mediate these cytokines’ signal transduction. One of the recently identified proteins, *JAK3*, is widely expressed in the epidermis and is essential for signaling many cytokine receptors, including IL-2R, IL-4R, IL-7R, and IL-9R [56]. Our results showed that the expression of *JAK3* mRNA was significantly reduced following treatment with SMV-FAPFs, which coincided with the increase of *CoTI*. Reduced expression of *JAK3* may be responsible for the reduced inflammation seen in SMV-FAPFs. Similarly, medicines that suppress *JAK3* expression have shown promising benefits in reducing psoriasis cutaneous inflammation [57]. Data shows that these novel SMV-FAPFs have markedly promoted wound healing compared to SMV or FA alone.

### 3.10. Comparative Histological Investigation

**Figure 13:**
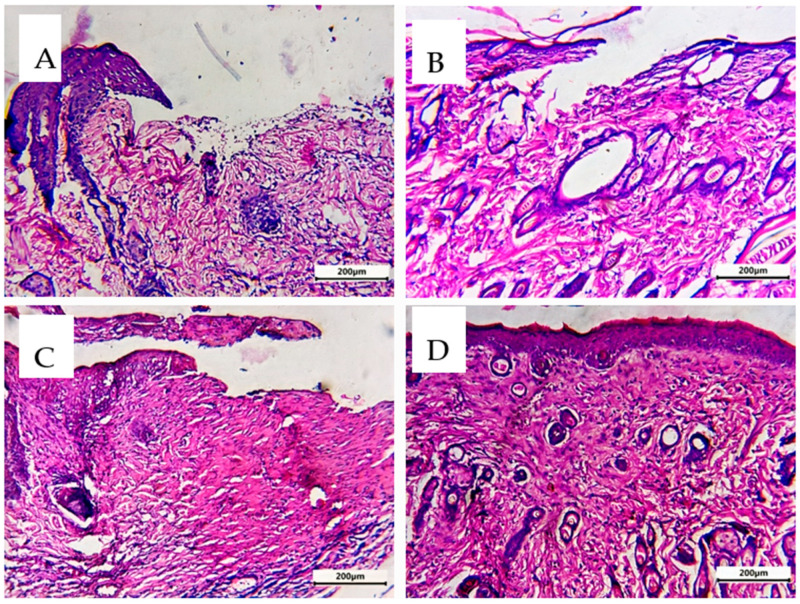
Sections of the thin skin of all group rats were stained with hematoxylin and eosin. (**A**) Untreated control group, (**B**) FA-treated group, (**C**) SMV-treated group, and (**D**) SMV-FAPFs-treated group.

Epidermis and dermis were both present in the analyzed rat skin. The epidermis consists of the four basal, spinous, granular, and cornified squamous keratinized epithelium layers. The dermo-epidermal junction (DED) is formed when epidermal invaginations and dermal papillae interdigitate. A highly vascular papillary layer surrounded a collagen, elastin, and connective tissue cell-rich reticular layer in the dermis [54]. In the untreated control group (A), the examined skin histopathological characteristics showed significant damage in the two layers of skin (epidermis and dermis) with loss of organization of structure. The treated skin with FA or SMV alone showed minimal or moderate improvements (B and C). Simvastatin, when applied topically, hastened the healing of wounds by promoting angiogenesis and lymphogenesis, and FA-treated wounds significantly accelerated tissue regeneration by promoting re-epithelialization, neo-vessel development, inflammatory cell migration, and collagen deposition. So, the combination of SMA and FA affected different stages of wound healing: inflammation, proliferation, and maturation, and resulted in more intact skin and less scar formation. Significant lesion healing and evident improvement in the skin layers (D) were observed in Group 4 receiving the combined treatment (Figure 13).

## 4. Conclusions

The solvent casting technique effectively produced SMV and FA-loaded chitosan dressings. The topical formulation in such dressings can significantly accelerate the rehabilitation process of infected wounds. The prepared sauces can be bent and molded to fit different wound sites, and they can be cut into varied sizes and shapes to provide different amounts of SMV. This in vivo wound reconstruction test on rats demonstrated conclusively that the combination of SMV and FA resulted in a more significant healing effect than that of SMV or FA alone. These combined films showed the highest expression of *CoTI*, four times more than the plain film, and the lowest expression of *JAK3*, which rapidly inhibited inflammation in treated wounds. Also, the histological analysis emphasized that the combination of SMV and CHT has a great healing power at different stages of the healing process. The curative effectiveness of SMV-FAPF dressings in managing the condition of chronic injuries will be the subject of additional studies.

## Figures and Tables

**Table 1:** Composition of different SMV-FAPFs.

	F1	F2	F3	F4	F5	F6	F7	F8	F9
SMV (mg)	20	20	20	20	20	20	20	20	20
EtOH/W ratio (% *v*/*v*)	10	10	10	20	20	20	30	30	30
Chitosan (% *w*/*v*)	0.5	1	1.5	0.5	1	1.5	0.5	1	1.5
Folic acid (mg)	40	40	40	40	40	40	40	40	40
Glycerol (% *v*/*v)*	1	1	1	1	1	1	1	1	1
Water to (mL)	20	20	20	20	20	20	20	20	20

EtOH/W ratio% level code: 10% (−1), 20% (0), and 30% (+1); CHT% level code: 0.5% (−1), 1% (0), and 1.5% (+1).

**Table 2:** Tensile strength, film expansion %, and cumulative % released at 3 h of SMV-FAPFs.

F. Code	TS (MPa)	Expansion %	Cumulative % Released at 3 h
F1	16.43 ± 0.92	41.32 ± 1.32	72.87 ± 2.32
F2	18.34 ± 0.87	43.65 ± 1.45	68. 24 ± 1.98
F3	19.84 ± 0.83	45.54 ± 1.67	64.54 ± 2.54
F4	14.65 ± 1.12	35.55 ± 1.98	62.76 ± 2.11
F5	15.55 ± 1.62	37.12 ± 1.55	59.43 ± 3.01
F6	16.97 ± 0.98	39.87 ± 1.39	55.33 ± 3.23
F7	11.21 ± 0.57	30.88 ± 1.24	52.98 ± 2.89
F8	12.34 ± 0.54	35.66 ± 1.66	50.43 ± 1.76
F9	14.39 ± 0.77	38.54 ± 1.77	46.44 ± 2.89

**Table 3:** Thickness, weight, drug content, surface pH, % moisture uptake, and mechanical (Tensile strength, expansion %, and folding endurance) characteristics for three selected SMV-FAPFs.

Formulation Code	F1	F2	F3
Thickness (μm)	165.45 ± 5.87	181.66 ± 4.39	187.98 ± 5.28
Weight 1 cm^2^ (mg)	7.7 ± 0.3	8.5 ± 0.2	9.3 ± 0.3
SMV content (*w*/*w*) %	9.65 ± 0.32	9.43 ± 0.35	9.24 ± 0.28
Surface pH	6.9 ± 0.19	7.2 ± 0.15	7.3 ± 0.14
Moisture uptake %	4.65 ± 0.43	4.52 ± 0.32	4.9 ± 0.54
Tensile strength (Mpa)	16.43 ± 0.92	18.34 ± 0.87	19.84 ± 0.83
Folding endurance	335 ± 4	367 ± 6	400 ± 7
Expansion %	41.32 ± 1.32	43.65 ± 1.45	45.54 ± 1.67

## Data Availability

The datasets generated during and analyzed during the current study are available from the corresponding authors upon reasonable request.
